# Joint Effects of Smoking and Sedentary Lifestyle on Lung Function in African Americans: The Jackson Heart Study Cohort

**DOI:** 10.3390/ijerph110201500

**Published:** 2014-01-28

**Authors:** Brenda W. Campbell Jenkins, Daniel F. Sarpong, Clifton Addison, Monique S. White, DeMarc A. Hickson, Wendy White, Cecil Burchfiel

**Affiliations:** 1 Jackson Heart Study, Jackson, Mississippi, MS 39213, USA; E-Mails: clifton.addison@jsums.edu (C.A.); wendywhite2001@yahoo.com (W.W.); 2 Xavier University, New Orleans, Louisiana, LA 70125, USA; E-mail: dsarpong@xula.edu; 3 Hinds Community College, Jackson, Mississippi, MS 39213, USA; E-Mail: mswhite427@yahoo.com; 4 My Brother’s Keepers, Jackson, Mississippi, MS 39157, USA; E-Mail: dhickson@mbk-inc.org; 5 Center for Disease Control and Prevention, Morgantown, West Virginia, WV 26505, USA; E-Mail:cecil.burchfiel@cdc.hhs.gov

**Keywords:** lung function, Jackson Heart Study, African Americans, smoking, sedentary lifestyle

## Abstract

This study examined: (a) differences in lung function between current and non current smokers who had sedentary lifestyles and non sedentary lifestyles and (b) the mediating effect of sedentary lifestyle on the association between smoking and lung function in African Americans. Sedentary lifestyle was defined as the lowest quartile of the total physical activity score. The results of linear and logistic regression analyses revealed that non smokers with non sedentary lifestyles had the highest level of lung function, and smokers with sedentary lifestyles had the lowest level. The female non-smokers with sedentary lifestyles had a significantly higher FEV1% predicted and FVC% predicted than smokers with non sedentary lifestyles (93.3% *vs.* 88.6%; p = 0.0102 and 92.1% *vs.* 86.9%; *p* = 0.0055 respectively). FEV1/FVC ratio for men was higher in non smokers with sedentary lifestyles than in smokers with non sedentary lifestyles (80.9 *vs.* 78.1; *p* = 0.0048). Though smoking is inversely associated with lung function, it seems to have a more deleterious effect than sedentary lifestyle on lung function. Physically active smokers had higher lung function than their non physically active counterparts.

## 1. Introduction

Previous studies have demonstrated that there is a correlation between smoking and lung function. Some researchers have concluded that forced expiratory volume in one second (FEV_1_) is a powerful predictor of general, pulmonary, and cardiovascular mortality and morbidity [1,2,3,4]. This is significant because the size of the airway, its elasticity, other respiratory disease, inflammation, and airway obstruction are physical characteristics of the pulmonary system that influence FEV_1_. Although, there is no current scientific evidence to explain the ethnic differences in lung function (LF), race and ethnicity have consistently been found to be important predictors of pulmonary function [5], suggesting that African Americans (AAs) have smaller lung volumes than whites [6]. Paucity of spirometry data from AAs might explain why early prediction equations for lung function parameters were developed exclusively among European Americans (EAs). Like smoking, sedentary lifestyle has been associated with less efficient pulmonary function. In a population known for a high prevalence of chronic diseases, there is a great need for a better understanding of the etiology of pulmonary dysfunction in AAs and increased knowledge of the predictors of increased risk. The results of several studies highlighted the need to further investigate sedentary lifestyle as a mediator between smoking and lung function. While Kaczynski *et al.* concluded that smoking and physical activity were largely incongruent behaviors [7], several other researchers have demonstrated that sedentary lifestyle and smoking are related to lung function. Exposure to heavy environmental tobacco smoke in an Oriental population was found to be associated with significant impairment of endothelial function, independent of age, gender, and traditional risk parameters. This may have adverse implications for cardiovascular risk in a modernizing Oriental population [8]. Ahmad *et al.* reported that physical inactivity and obesity can impair FVC and FEV1, while appropriate aerobic exercise training can partly improve FVC and FEV1 due to the respiratory muscle performance enhancement [9]. Garcia-Aymerich *et al.* found that moderate to high levels of regular physical activity are associated with reduced lung function decline and COPD risk among smokers [10]. In a study in 2011 by Katz *et al.*, it was found that physically inactive individuals with obstructive lung disease (OLD) had increased odds of increase in disability after controlling for baseline disability, lung function, and other covariates [11]. These results provide strong support for the importance of maintaining physical activity among individuals with obstructive disease. In addition, Jakes *et al.* reported that physical activity is associated with higher levels of FEV(1), whereas sedentary behavior, like television viewing, is associated with lower levels [12].

Since smoking and physical activity are largely incongruent behaviors [7], and the literature is limited in its accounting of the interplay of smoking and sedentary lifestyle in relation to lung function in AAs, this study was conducted to examine the joint effects of smoking and sedentary lifestyle of AAs using the Jackson Heart Study (JHS) data. In addition, it was decided to examine whether sedentary lifestyle mediate the relationship between smoking and lung function in AAs in the JHS.

### 1.1. Smoking and Lung Function

Early life involvement in smoking during childhood might prevent the lung from attaining complete development and increase chances of illnesses. Approximately 20% of all annual deaths [13,14], and 157 billion dollars in US health care expenses [15] are attributable to cigarette smoking. Although AAs are more likely to have smoked fewer cigarettes per day [16] or begin smoking later in life (tantamount to fewer pack years) [17], they are disproportionately affected by smoking related diseases [16,17].

### 1.2. Sedentary Lifestyle and Lung Function

Two thirds of US adults do not meet the current recommendation of at least 30 minutes of moderate (physical activity on 5–7 days per week) [13]. Higher prevalence of physical inactivity among AAs might account for elevated morbidity and mortality rates [12,14,15,16]. AA women compared to men have markedly low levels of physical activity [17]. In addition, airflow limitation resulting from sedentary lifestyle is an independent predictor of future cardiovascular events in patients with various cardiovascular risk factors [18]. Although there are known associations between lung function, physical activity, and disease [19,20], research supporting the association between physical activity and lung function is scarce [21,22,23,24,25,26,27,28,29], and the mechanisms by which physical inactivity might influence FEV_1_ are unclear.

### 1.3. Significance

Low levels of physical activity have been reported among AAs compared to EAs, as have negative associations between (a) sedentary lifestyle and lung function and (b) between smoking and physical activity [13,30]. This study sought to examine if smoking has a negative impact on lung function and cardiovascular health and to investigate the mediating role of smoking and sedentary lifestyle on health status. Specifically, the study addressed the following questions:

1. Are there differences in the lung function of smokers and non-smokers who are physically active *versus* smokers and non-smokers who are not physically active?
2. Does sedentary lifestyle mediate the relationship between smoking and lung function in AAs in the JHS?

## 2. Design/Methods

### 2.1. Study Population

The JHS is the largest single-site, population-based cohort study of cardiovascular disease (CVD) among AAs in the U.S. The study sample consists of AA adults aged 21–95 residing in urban and rural areas in the tri-county region: Hinds, Madison, and Rankin Counties, which constitutes the Jackson, Mississippi metropolitan statistical area (MSA) [31]. The JHS cohort included 5,301 participants (mean age = 55.6, SD = 12.7, 63.3% women), equivalent to 7% of all African Americans aged 21–95 residing in the Jackson MSA [31]. Details of the study design and recruitment protocol have been described elsewhere [31,32,33,34,35,36,37,38]. The analytic sample size was 3256 of the 5301 based on JHS Exam 1 data (2000–2004). This sample excluded the following: (1) Prevalent CVD cases (n = 545); (2) self-reported physician diagnosed asthma (n = 729); and (3) missing values for; (a) lung function measures (n = 185); (b) socio-demographic factors (n = 586). For the analytic sample, the mean age was 54 years (SD = 13) ranging from 21–93 years, with 64% of the sample comprised of women.

### 2.2. Outcome Measures

The following lung function measures represented the study outcome measures: (1) Forced expiratory volume in one second (FEV_1_); (2) forced vital capacity (FVC); (3) FEV_1_/FVC ratio; (4) percent predicted FEV_1_ (PPFEV_1_); (5) percent predicted FVC (PPFVC); and (6) airway obstruction. Pulmonary function (PF) was measured using computerized spirometry; maximum values of FVC and FEV_1_ were selected for analysis based on recommendations from the American Thoracic Society [28]. Derivation of the percent predicted measures of pulmonary function measures were based on the National Health and Nutrition Examination Survey (NHANES) and were formulated based on the race/ethnicity, height, age and sex of the participant [39].

### 2.3. Independent Variables

*Smoking Status*—Current cigarette smoking (CCS) was defined as a positive response to “Have you smoked more than 400 cigarettes in your lifetime?” and “Do you now smoke cigarettes”. Former cigarette smoking was defined as a positive response to the first question and a negative response to the latter question. Never smokers are those who responded no to the first question.

*Sedentary lifestyle (SL)* was defined as the lowest quartile of the total physical activity score (TPAS); 3 ≤ TPAS < 6.5. Participants classified as not having sedentary lifestyle had a TPAS in the upper 75th percentile. The TPAS is a summary score of the intensity, frequency, and duration of various physical activities derived from the 30-item Jackson Heart Physical Activity Cohort (JPAC) validated interviewer-administered survey that assesses physical activity over the past 12 months [33]. The survey took 15 minutes to administer [33]. TPAS which ranges from 3 (low) to 16.9 (high) was validated against results from 24-hour accelerometer and pedometer monitoring.

The two independent measures were stratified to derive four study groups defined by participants who were (a) non-current smokers and had non-sedentary lifestyle (NSK_NSL), (b) non-current smokers and had sedentary lifestyle (NSK_SL), (c) current smokers who had non-sedentary lifestyle (SK_NSL), and (d) current smokers who had sedentary lifestyle (SK_SL). *Gender* was treated as an effect modifier because a number of statistically significant gender heterogeneity was observed from prior work showing differential association of physical activity and smoking with lung function outcomes by gender.

### 2.4. Covariates

Researchers believe that some of the differences noticed in lung function may be attributed to body composition [18,40,41,42,43] dietary intake, physical activity, socioeconomic factors [18,41], age [44], and genetics [40,41,42,43,44]. Factors adjusted for in this analysis were: Age, educational attainment, marital status, family income, body mass index, total dietary fat, fiber and carbohydrate, alcohol consumption in the past 12 months, history of hypertension, anti-hypertensive therapy, history of type 2 diabetes, anti-diabetic medication, supplement use, and cumulative pack years (*i.e.*, average number of cigarettes smoked per day, divided by 20 and multiplied by the number of years smoked) as an indicator of smoking status. Duration of smoking was computed as the difference between the age at which smoking was initiated and the age at baseline examination. Intake of dietary fat, fiber, and carbohydrate was determined from participant responses to the JHS food frequency short-form questionnaire developed in conjunction with the Human Nutrition Research Center on Aging at Tufts University and the Delta Nutrition Intervention Research Initiative [35].

### 2.5. Statistical Analysis

Descriptive statistics were used to describe the characteristics of the study sample and the observed lung function measures across the four study groups. General Linear models (GLM) were used to compare observed lung function measurement across the four study groups and the potential covariates. Logistic regression analysis was used to test for association of categorical covariates and the smoking-sedentary lifestyle groupings. GLM (via SAS PROC GENMOD) was used also to assess the differences in the lung function of smokers who are physically active *versus* non-smokers who are not physically active. Linear or logistic regression was used to test the mediation of sedentary lifestyle on the relationship between smoking and lung function in African Americans. All inferential statistics were stratified by gender. The significance level for all statistical tests was 0.05.

## 3. Results

**Figure 1:**
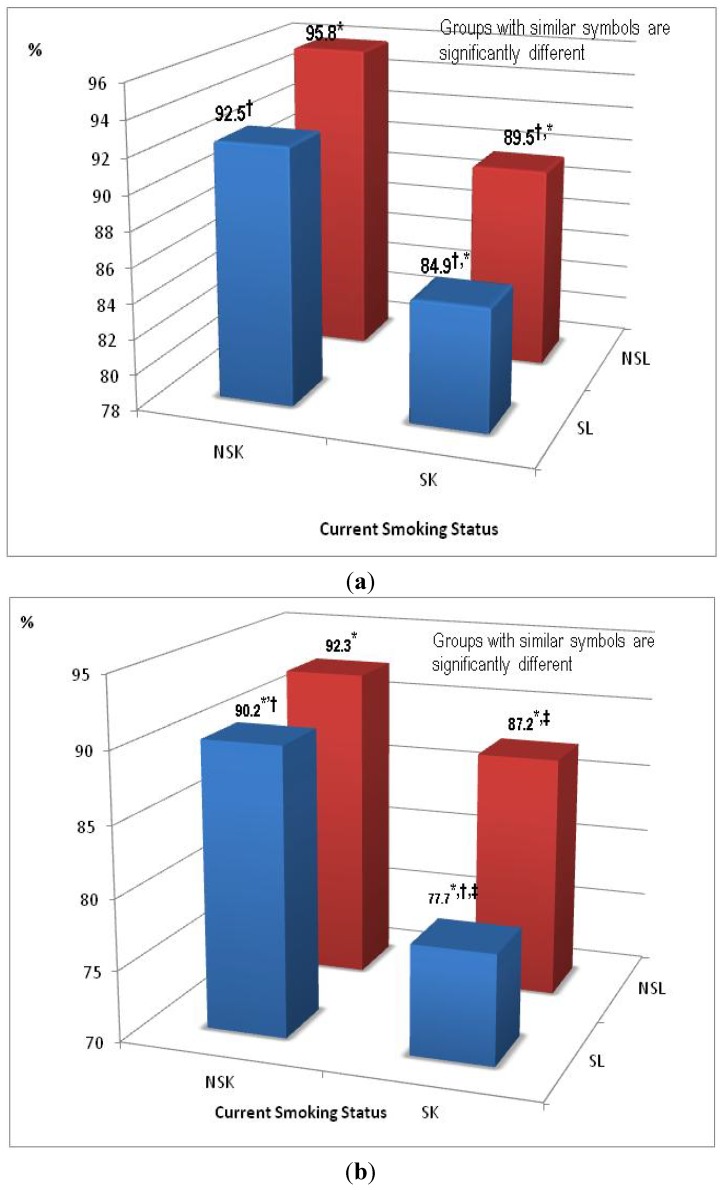
(**a**) Age-adjusted Means of FEV1 % Predicted for Women (n = 2,065). (**b**) Age-adjusted Means of FEV1 % Predicted for Men (n = 1,191).

Unadjusted sex-stratified comparative analysis of measures of lung function (FEV_1,_ FVC, FEV_1_/FVC ratio, PPFEV_1_, PPFVC and airway obstruction) indicated significant differences between the four subgroups for both genders. In general, for both genders, participants who were non smokers with a non sedentary lifestyle (NSK-NSL) group had the highest level of lung function and participants who were smokers and had a sedentary lifestyle (SK-SL) had the lowest lung function level. Figure 1a,b graphically depict differences in the age-adjusted mean PPFEV_1_ (single measure of lung function) values between the four groups. Although participants who were non smokers and had a sedentary lifestyle (NSK_SL = 92.5%) had slightly higher lung function levels than smokers with a non sedentary lifestyle (SK_NSL = 89.5%), the difference was not statistically significant.

Of the 2,065 women studied, 1,425 (69.0%) were classified as non-current smokers with a non-sedentary lifestyle (NSK-NSL), 440 (21.3%) were classified as non-current smokers with a sedentary lifestyle (NSK-SL); 152 (7.4%) were current smokers with a non-sedentary lifestyle (SK-NSL); and 48 (2.3%) were current smokers with a sedentary lifestyle (SK-SL). Similarly, 826 (69.4%) of the total 1,191 men were non smokers with non sedentary lifestyles (NSK-NSL), 167 (14.0%) were non smokers with sedentary lifestyles (NSK-SL); 155 (13.0%) were smokers with non sedentary lifestyles (SK-NSL); and 43 (3.6%) were smokers with sedentary lifestyles (SK-SL). There were significant differences across the four subgroups (smoking/sedentary lifestyle groups) for the following characteristics of the study sample for both men and women: age, BMI, family income, education, and alcohol consumptions. The use of anti- hypertension and diabetes therapy differed across the four groups; this pattern was similar across gender. Men who were current smokers, irrespective of sedentary lifestyle had greater mean total dietary fiber intake compared to the non-current smokers; this was also true for women (See Table 1).

**Table 1:** Sex-stratified socio-demographic, lifestyle, cardiovascular, and lung function characteristics by smoking and sedentary lifestyle group.

Characteristics	NSK_NSL	NSK_SL	SK_NSL	SK_SL	*p*
Women (n = 2,065)	(n = 1,425)	(n = 440)	(n = 152)	(n = 48)	
Age, years ^†^	52 ± 12	62 ± 12	51 ± 11	56 ± 9	****
Body Mass index	32.6 ± 7.5	33.2 ± 7.7	31.4 ± 7.3	31.1 ± 7.4	*
Marital status, Married (%)	46.5	47.1	42.1	35.4	NS
Family income, Affluent (%)	31.2	18.6	18.4	12.5	****
Education, ≥College Associate (%)	51.0	30.7	29.6	27.1	****
Alcohol Use (%)	43.6	23.1	65.1	54.2	****
Hypertension (%)	56.5	73.7	49.3	78.7	****
Anti-hypertensive therapy (%)	49.1	67.4	38.9	65.2	****
Type 2 Diabetes Mellitus (%)	14.8	24.9	11.5	28.3	****
Anti-diabetic medication (%)	12.9	23.3	8.2	27.3	****
Total dietary fiber, grams	15.0 ± 6.8	13.8 ± 5.8	16.6 ± 7.4	14.9 ± 6.8	***
FEV_1_^†^	2.21 ± 0.48	1.90 ± 0.46	2.14 ± 0.50	1.89 ± 0.44	****
FVC ^†^	2.70 ± 0.57	2.38 ± 0.58	2.64 ± 0.58	2.45 ± 0.53	****
FEV_1_/FVC ^†^	82.1 ± 8.5	80.2 ± 8.8	81.3 ± 9.1	77.4 ± 9.2	****
FEV_1_% Predicted ^†^	95.4 ± 16.7	93.8 ± 19.6	89.0 ± 15.5	85.2 ± 16.1	****
FVC% Predicted ^†^	93.7 ± 17.0	92.4 ± 20.8	88.6 ± 15.5	87.8 ± 15.1	**
Airway Obstruction (%)	8.1	10.7	10.1	18.8	*
Men (n = 1,191)	(n = 826)	(n = 167)	(n = 155)	(n = 43)	
Age, years ^†^	52 ± 12	63 ± 12	49 ± 11	61 ± 11	****
Body Mass index ^†^	29.9 ± 5.9	30.2 ± 6.0	28.0 ± 6.3	28.1 ± 8.4	***
Marital status, Married (%)	75.7	75.3	58.7	46.5	****
Family income, Affluent (%)	45.2	28.1	28.4	23.3	****
Education, ≥College Associate (%)	48.1	25.8	27.1	16.3	****
Alcohol Use (%)	60.1	45.5	87.1	67.4	****
Hypertension (%)	53.2	74.7	48.7	62.8	****
Anti-hypertensive therapy (%)	41.5	57.4	22.8	47.5	****
Type 2 Diabetes Mellitus (%)	12.3	22.4	9.2	23.8	***
Anti-diabetic medication (%)	10.8	20.7	6.8	17.1	****
Total dietary fiber, grams	17.8 ± 8.3	16.4 ± 6.7	21.0 ± 10.0	21.4 ± 16.7	****
FEV_1_^†^	3.07 ± 0.66	2.59 ± 0.63	2.97 ± 0.66	2.30 ± 0.69	****
FVC ^†`^	3.80 ± 0.78	3.32 ± 0.92	3.84 ± 0.74	3.09 ± 0.77	****
FEV_1_/FVC ^†^	81.1 ± 8.1	78.8 ± 10.4	77.5 ± 10.0	74.5 ± 12.0	****
FEV_1_% Predicted ^†^	92.6 ± 15.8	88.7 ± 17.2	87.9 ± 15.3	76.4 ± 17.8	****
FVC% Predicted	91.1 ± 15.9	88.3 ± 27.4	90.8 ± 14.1	79.8 ± 17.3	***
Airway Obstruction (%)	11.5	18.1	18.7	41.9	***

Note: ^†^ mean ± SD; P-values associated with mean-difference between the four groups; NS: Non-significant; *: *p* < 0.05, **: *p* < 0.01, ***: *p* < 0.001, ****: *p* < 0.0001; NSK_NSL: participants who were non current smokers and had non-sedentary lifestyle; NSK_SL: participants who were non current smokers and had sedentary lifestyle; SK_NSL: participants who were current smokers and had non-sedentary lifestyle; and SK_SL: participants who were current smokers and had sedentary lifestyle.

Table 2 provides a summary of the sex-stratified analysis of participants in the non-smoking/sedentary lifestyle (NSK_SL) and smoking/non sedentary lifestyle (SK_NSL) groups. Focusing on the multivariable adjusted models (model 2) in women, PPFEV_1_ was significantly higher in the non-smoking/sedentary lifestyle group (NSK_SL) than in the smoking/non sedentary lifestyle group (SK_NSL) (93.3% *vs.* 88.6%; *p* = 0.0102). Similarly, PPFVC was higher in the non-smoking/ sedentary group (NSK_SL) than in the smoking/non sedentary lifestyle group (SK_NSL) (92.1% *vs.* 86.9%; *p* = 0.0055). However, for the men, FEV_1_/FVC ratio was higher in the non-smokers/sedentary group (NSK_SL) than in the smoking/ non sedentary lifestyle group (SK_NSL) (80.9 *vs.* 78.1; *p* = 0.0048). However, significant difference between the two groups for the FVC in the age-adjusted model (Model 1) was attenuated for men and not for women when adjustment for additional covariates was performed.

**Table 2:** Sex-stratified comparative analysis of lung function measure among smokers without sedentary lifestyle *vs.* non-smokers with sedentary lifestyle.

Characteristics	Model 1			Model 2		
	NSK-SL	SK-NSL	*p*	NSK-SL	SK-NSL	*p*
Women						
Forced expiratory volume in 1 second (FEV_1_)	2.08 ± 0.02	2.07 ± 0.03	0.7342	2.11 ± 0.02	2.05 ± 0.04	0.1932
Forced Vital Capacity (FVC)	2.56 ± 0.03	2.57 ± 0.04	0.8300	2.59 ± 0.03	2.51 ± 0.05	0.1355
FEV_1_/FVC Ratio	81.6 ± 0.42	80.7 ± 0.68	0.2670	81.5 ± 0.46	81.6 ± 0.76	0.9269
FEV1% Predicted	92.5 ± 0.86	89.5 ± 1.40	0.0689	93.3 ± 0.92	88.6 ± 1.53	0.0102
FVC % Predicted	91.2 ± 0.89	89.1 ± 1.44	0.2028	92.1 ± 0.95	86.9 ± 1.58	0.0055
Airways Obstruction	1.00	0.59 (0.30,1.15)	0.1200	1.00	0.68 (0.32,1.48)	0.3328
Men						
FEV1	2.85 ± 0.04	2.86 ± 0.05	0.9037	2.91 ± 0.05	2.89 ± 0.05	0.7679
FVC	3.56 ± 0.06	3.74 ± 0.06	0.0333	3.59 ± 0.06	3.71 ± 0.06	0.1543
FEV1/FVC	80.8 ± 0.68	76.6 ± 0.69	<0.0001	80.9 ± 0.71	78.1 ± 0.69	0.0048
FEV1% Predicted	90.2 ± 1.28	87.2 ± 1.29	0.1042	91.2 ± 1.37	87.5 ± 1.34	0.0641
FVC % Predicted	89.6 ± 1.43	90.2 ± 1.43	0.7942	89.2 ± 1.32	89.0 ± 1.29	0.9312
Airways Obstruction	1.00	1.21 (0.62, 2.34)	0.5744	1.00	1.46 (0.66, 3.24)	0.3471

Note: Model 1—Age-adjusted model; Model 2—Model 1 plus marital status, income, body mass index, alcohol consumption, prevalent hypertension, anti-hypertension medication, prevalent type 2 diabetes, anti-diabetic medication, smoking duration and inhalation pattern.

In Table 3 and Table 4, using the multivariable-adjusted model (Model 3), the following findings were observed for both genders. Smoking status was significantly associated with FEV_1_, FVC, PPFEV_1_, and PPFVC. After accounting for sedentary lifestyle (Model 4), smoking status was still significantly associated with FEV_1_, PPFVC, and PPFVC for both genders. For the four above mentioned lung function measures, sedentary lifestyle was significantly associated with FEV_1_ and PPFEV_1_ for both genders. Additionally, for the men, sedentary lifestyle was significantly associated with FVC (*p* = 0.0019), but not PPFVC (*p* = 0.1379). Comparisons of Models 3 and 4 for both genders revealed that adjustment for physical activity did not alter the associations between smoking status and lung function, thus indicating minimal evidence of mediation by physical activity.

**Table 3:** Multivariate and Odds Ratio test of mediation of sedentary lifestyle on the association of smoking status and lung function among African American women.

Lung Functioning Measures	Model 1	Model 2	Model 3	Model 4
**1. Bivariate and Multivariable Analysis: FEV_1_**				
*Smoking status:*				
Never	2.13	2.11	2.12	2.11
Former	2.18	2.15	2.22	2.20
Current	2.03	2.01	2.06	2.05
*p-value*	0.0002	0.0005	0.0005	0.0011
*Physical Activity:*				
Non-Sedentary Lifestyles	--	2.13	--	2.15
Sedentary Lifestyles	--	2.05	--	2.09
*p-value*	--	0.0002	--	0.0215
**FVC**				
*Smoking status:*				
Never	2.61	2.59	2.63	2.62
Former	2.71	2.68	2.66	2.64
Current	2.55	2.53	2.46	2.45
*p-value*	0.0015	0.0039	0.0004	0.0007
*Physical Activity:*				
Non-Sedentary Lifestyles	--	2.64	--	2.59
Sedentary Lifestyles	--	2.56	--	2.55
*p-value*	--	0.0031	--	0.1246
**FEV_1_/FVC**				
*Smoking status:*				
Never	81.7	81.6	80.8	80.7
Former	81.2	81.1	83.9	83.7
Current	80.0	79.9	83.6	83.5
*P-value*	0.0161	0.0156	0.1493	0.1616
*Physical Activity:*				
Non-Sedentary Lifestyles	--	81.1	--	82.8
Sedentary Lifestyles	--	80.7	--	82.4
*P-value*	--	0.3752	--	0.3687
**Percent Predicted FEV_1_**				
*Smoking status:*				
Never	94.9	94.1	94.2	93.6
Former	95.4	94.2	97.7	96.8
Current	88.3	87.6	90.2	89.6
*P-value*	<0.0001	<0.0001	<0.0001	0.0002
*Physical Activity:*				
Non-Sedentary Lifestyles	--	93.6	--	94.5
Sedentary Lifestyles	--	90.3	--	92.2
*P-value*	--	0.0005	--	0.0216
**Percent predicted FVC**				
*Smoking status:*				
Never	93.1	92.5	93.6	93.3
Former	94.4	93.5	93.4	92.8
Current	88.6	88.0	85.8	85.4
*P-value*	0.0008	0.0013	<0.0001	0.0001
*Physical Activity:*				
Non-Sedentary Lifestyles	--	92.6	--	91.3
Sedentary Lifestyles	--	90.1	--	89.7
*P-value*	--	0.0096	--	0.1312
**Percent Predicted FEV_1_/FVC**				
*Smoking status:*				
Never	98.9	99.2	100.1	100.6
Former	99.9	100.3	96.9	97.6
Current	101.1	101.4	96.6	97.0
*P-value*	0.6568	0.6468	0.8819	0.08862
*Physical Activity:*				
Non-Sedentary Lifestyles	--	99.7	--	97.5
Sedentary Lifestyles	--	101.0	--	99.3
*P-value*	--	0.5081	--	0.3954
**2. Odds Ratio Analysis** **Obstruction**				
*Smoking status:*(Ref: Never)				
Former	0.80 (0.51,1.33)	0.88 (0.54,1.42)	1.18 (0.31,4.39)	1.33 (0.36,4.96)
Current	1.36 (0.86,2.16)	1.34 (0.84,2.13)	1.87 (0.50,6.98)	1.93 (0.52,7.21)
*Physical Activity(Ref: Non-Sedentary Lifestyles*				
Sedentary Lifestyles	--	1.90 (1.33,2.73)	--	1.84 (1.25,2.71)

Note: Model 1 – Age – Sex – Adjusted: Model 2 – Age – Sex – Adjusted + Smoking (Never, Former and Current) + Physical Activity (Sedentary lifestyles), Model 3 – multivariable adjusted + Smoking (Never, Former and Current), Model 4 - multivariable adjusted + Smoking (Never, Former and Current) + Physical Activity (Sedentary Lifestyles); “--”: denotes no values were obtained because Sedentary Lifestyles was not includes in the model.

**Table 4:** Multivariable and Odds ratio Analysis of mediation of sedentary lifestyle on the association of smoking status and lung function among African American men.

Lung Functioning Measures	Model 1	Model 2	Model 3	Model 4
**1. Bivariate and Multivariable Analysis: FEV_1_**				
*Smoking status:*				
Never	2.98	2.91	2.89	2.84
Former	3.04	2.97	3.20	3.13
Current	2.79	2.73	2.97	2.91
*P-value*	<0.0001	<0.0001	0.0002	0.0003
*Physical Activity:*				
Non-Sedentary Lifestyles	--	2.97	--	3.04
Sedentary Lifestyles	--	2.77	--	2.88
*P-value*	--	<0.0001	--	0.0007
**FVC**				
*Smoking status:*				
Never	3.69	3.61	3.60	3.54
Former	3.82	3.73	4.01	3.93
Current	3.64	3.57	3.77	3.70
*P-value*	0.0220	0.0406	0.0013	0.0019
*Physical Activity:*				
Non-Sedentary Lifestyles	--	3.76	--	3.82
Sedentary Lifestyles	--	3.52	--	3.62
*P-value*	--	<0.0001	--	0.0021
**FEV_1_/FVC**				
*Smoking status:*				
Never	81.1	81.1	79.8	79.7
Former	79.9	79.9	81.3	81.2
Current	76.6	76.6	80.1	80.0
*P-value*	<0.0001	<0.0001	0.3023	0.3071
*Physical Activity:*				
Non-Sedentary Lifestyles	--	79.2	--	80.4
Sedentary Lifestyles	--	79.1	--	80.2
*P-value*	--	0.9491	--	0.8139
**Percent Predicted FEV_1_**				
*Smoking status:*				
Never	91.8	90.7	87.6	86.8
Former	92.3	91.1	99.2	98.0
Current	85.1	84.2	92.7	91.6
*P-value*	<0.0001	<0.0001	<0.0001	<0.0001
*Physical Activity*				
Non-Sedentary Lifestyles	--	90.4	--	93.6
Sedentary Lifestyles	--	86.9	--	90.6
*P-value*	--	0.0066	--	0.0294
**Percent predicted FVC**				
*Smoking status:*				
Never	90.1	89.2	86.5	85.8
Former	92.2	91.3	99.0	98.1
Current	88.1	87.4	92.7	91.9
*P-value*	0.0513	0.0704	0.0001	0.0002
*Physical Activity:*				
Non-Sedentary Lifestyles	--	90.6	--	93.1
Sedentary Lifestyles	--	87.9	--	90.8
*P-value*	--	0.0625	--	0.1379
**Percent Predicted FEV_1_/FVC**				
*Smoking status:*				
Never	99.0	99.1	100.9	101.1
Former	100.2	100.3	98.3	98.6
Current	105.4	105.5	99.4	99.7
*P-value*	<0.0001	<0.0001	0.5993	0.6157
*Physical Activity:*				
Non-Sedentary Lifestyles	--	101.5	--	99.4
Sedentary Lifestyles	--	101.9	--	100.2
*P-value*	--	0.7670	--	0.5635
**2. Odds Ratio Analysis Obstruction**				
*Smoking status(Ref: Never)*				
Former	1.18 (0.78,1.79)	1.21 (0.80,	0.88 (0.11,1.38)	0.41 (0.11,1.47)
Current	2.38 (1.60,3.56)	2.31 (1.54,3.46)	0.75 (0.21,2.72)	0.78 (0.21,2.84)
*Physical Activity: (Ref: Non-Sedentary Lifestyles)*				
Sedentary Lifestyles	--	1.71 (1.14,2.57)	--	1.62 (1.03,2.55)

Note: Model 1—Age – Sex – Adjusted; Model 2—Age – Sex – Adjusted + Smoking (Never, Former and Current) + Physical Activity (Sedentary lifestyles); Model 3—multivariable adjusted + Smoking (Never, Former and Current); Model 4—multivariable adjusted + Smoking (Never, Former and Current) + Physical Activity (Sedentary Lifestyles); “--”: denotes no values were obtained because Sedentary Lifestyles was not includes in the model.

## 4. Discussion

The Jackson Heart Study collected pulmonary function data on over 5,000 AA adults of varied socioeconomic status. Because of the paucity of pulmonary function studies involving African American adults, this study was designed to examine several measures of lung function in relation to smoking practices and physical activity among an African American population. An examination of the data leads to the conclusion that African American men and women who are free of smoking and are physically active have the best lung function and those who smoke and have a sedentary lifestyle have the worst lung function. In short, the analysis revealed that persons who smoked and were physically active had better lung function than those who were not physically active. These findings are consistent with some, but not all previous investigations. Some research studies have purported that there are associations between lung function, physical activity, and disease [19,20]. However, research supporting the association between physical activity and lung function is scarce [21,22,23,24,25,26,27,28,29], and the mechanisms by which physical inactivity might influence FEV_1_ are unclear.

Covariates were also included in these analyses because the spirometric variables are believed to be influenced by a number of external and internal factors. Although there were significant differences between the four subgroups, the patterns of difference were different for men and women. Those who were in the smoking/sedentary lifestyle group had the highest prevalence of hypertension and anti-hypertensive medication usage. For men, those who were in the non smoking/sedentary lifestyle group had the largest percentage of hypertension and anti-hypertensive medication use. However, for diabetes and anti-diabetic therapy, although there was significant difference between the four subgroups with men and women in the smoking/ sedentary lifestyle group having the largest percentage, the men had a consistently lower percentage of diabetes and use of anti-diabetic medication across the four subgroups. Men and women who were current smokers, irrespective of sedentary lifestyle, indicated a greater mean total dietary fiber intake compared to the non-current smokers.

For both genders, all of the unadjusted lung function measures differed significantly across the four study groups. However, these differences were attenuated for a number of the lung function measures in the multivariable adjusted models, suggesting that the differences initially observed were due to differences in the potential confounders and the co-morbidities among the four study groups and supporting the belief that the spirometric variables could be influenced by a number of external and internal factors. Also, the study revealed that for the multivariable adjusted models, there were gender differences in the significant findings among the set of measures of lung function between smokers who had a non-sedentary lifestyle and non-smokers who had a sedentary lifestyle. Others have found that body composition [18,41,42,43,44], dietary intake, physical activity, socioeconomic factors [18,44], age [44], and even genetics [40,43,44] may explain differences in lung function. Given that FEV_1_ has been documented as a powerful predictor of general, pulmonary, and cardiovascular mortality and morbidity[1,2,3,4], if we were to use FEV_1_ and other derivatives of it (FEV_1_ % Predicted and FEV_1_/FVC ratio), then the following inferences could be made based on the results of this study:

1. Women who are nonsmokers and have a sedentary lifestyle tend to have a significantly higher level of lung function (FEV_1_ % Predicted) compared to women smokers who are non-sedentary.
2. Men who are nonsmokers and have a sedentary lifestyle tend to have a significantly higher level of lung function using the FEV_1_/FVC ratio as a measure of lung function.

There is a statistically significant difference for women non smokers when using the FEV_1_ values for lung function and for men non smokers when using the FEV_1_/FVC values. The difference in outcome measure may attach some additional limitations to these findings. While the outcome measure for lung function for men and women for which significance was noted is not identical, the finding is important enough to warrant concern and establish the need to develop tools to improve or maintain the pulmonary function of smokers and/or individuals prone to developing pulmonary pathologies. The aim of this investigation was to examine smoking and sedentary lifestyle as possible factors associated with deficiency in lung function. Based on these findings, the need to develop a primary preventive strategy to help prevent smokers from developing pulmonary diseases could be an implication of this research. These results could raise concerns and provide clinicians, patients and healthy with additional information as the struggle continues to reduce the prevalence of chronic disease, to improve pulmonary function, health status, and a sense of well-being among African Americans.

Previous research has demonstrated that there is racial/ethnic difference in the prevalence of smoking in the US [45], and the results from this study are consistent with the belief that smoking as a risk factor for cardiovascular disease is not a major issue among the African American population. The percentages of current smokers were relatively low for men (16.6%) and women (9.7%). The percentage of men and women who were smokers and had a sedentary lifestyle was also very low; 2.3% and 3.6%, respectively.

The patterns of the joint effects of smoking and sedentary lifestyle on lung function vary with the various measures of lung function and are not consistent across gender. However, the results of this study provide evidence that is consistent with the postulates of Garcia-Aymerich *et al.* (2006) that regular physical activity modifies smoking-related lung function decline and reduces risk of chronic obstructive pulmonary disease [10]. These findings underscore that smoking and sedentary lifestyle both have deleterious effects on lung function in African Americans with some evidence to suggest that sedentary lifestyle is the least harmful. The interaction between physical activity and smoking should be accounted for when estimating the burden of respiratory disease. For both males and females, being a smoker who is physically active does not necessarily translate to increased lung function. A smoke free lifestyle more than a physically active lifestyle seems to dictate increased lung function. Respiratory function was tested using spirometric measures which were examined to assess the influence of smoking and sedentary lifestyle on lung function. Six spirometric variables were used to examine the pulmonary function and evaluate differences between groups. Non smokers who were not physically active (sedentary) displayed significantly higher values of forced expiratory volume in one second (FEV1) and forced vital capacity (FVC) than smokers and participants who were non sedentary (physically active). Non-smokers had better lung capacity than smokers, regardless of whether the smokers were physically active. That was true for both males and females. Living a non sedentary lifestyle, which involves engagement in physical activity and not smoking, is associated with maintenance of respiratory fitness and lung function. It can be assumed then that changing physical activity habits to ensure a non sedentary lifestyle will lead to improved lung function.

One question that comes to mind is “can physical activity by itself improve lung function?” This study shows that smokers who are non sedentary exhibit some degree of lung-function impairment compared to nonsmokers, but still have better lung function than smokers who are sedentary. Smoking can be viewed as a catalyst for increasing respiratory symptoms and diminished lung function. The lower FEV1 and FVC values of the smokers compared to nonsmoker groups could be attributed to possible airways obstruction which could mean that smokers had developed some degree of lung function impairment compared to non-smoker.

Elimination of smoking and incorporating physically active lifestyles can help to increase respiratory capacity. This is important information for use with health promotion and health education programs that are geared towards reducing the negative effects of smoking as one of the main risk factors for chronic diseases, such as cancer, lung diseases, cardiovascular diseases, and the cardio-respiratory functions. The significant differences between smokers and nonsmokers could be explained with the functional and structural abnormalities that smoke cause on terminal bronchioles (mucus plugs, accumulation of pigment laden macrophages, goblet and squamous cells metaplasia, ulceration, inflammatory cell infiltrate, smooth muscle hypertrophy, fibrosis and excessive pigments [46].

The present investigation showed the effects of smoking on lung function and the influence of physical activity on increasing respiratory abilities. The values of spirometric variables presented in this study for the smokers and non-smokers could be important for assessing future lung function status and detection of potential obstructive pulmonary diseases by evaluating and estimating the influence of smoking. This study examined various combinations of smoking and sedentary lifestyles as they relate to lung function in the African American cohort in the Jackson Heart Study. It would be prudent to encourage participation in physical activity, while modifying smoking habits as effective strategies for African American populations to reduce preventable risk factors, limit health disparities, and improve health status. Exercise and smoking elimination are important practices for promoting pulmonary health.

Given the paucity of pulmonary function data in African Americans and given the variability in significant findings for the various measures of lung function used in this study across gender, it is important that more large epidemiological studies on lung function be conducted in African American populations. Also, longitudinal data are needed to examine causal relations and to determine whether changes in smoking status and sedentary behavior lead to changes in lung function. In summary, smoking and physical inactivity both negatively impact lung function, with smoking having a more deleterious effect. This finding is important given that cigarette smoking is responsible for 440,000 deaths annually and costs $157 billion dollars in health care expenses, accounting for 20% of all deaths in the United States, along with several diseases, and other adverse health conditions [15].

### Study Limitations and Strengths

Given that the JHS sample was limited to adults and was not designed to be a nationally representative sample, findings from the study might not be generalized to all African Americans. Low percentages of current men (16.6%) and women (9.7%) smokers resulted in small sample sizes for the smoking/sedentary group for men (n = 43; 3.6% of total men sample) and women (n = 48; 2.3% of total women sample). Lack of a standard definition of sedentary lifestyle is a limitation and raises questions as to whether or not the lowest quartile of the TPAS adequately reflects a sedentary lifestyle. This issue is not unique to this study since the task of coming up with an international standard definition for sedentary lifestyle is difficult [39]. In fact, exploring different measures of sedentary lifestyle in the same manner that lung function was evaluated using several measures could eventually lead to a universally acceptable definition of sedentary lifestyle. It might be useful in future studies if physical activity would be assessed using more categories such as low, moderate and high.

## 5. Conclusions

In this cross sectional study, after controlling for covariates, being a non smoker and being active were found to be associated with better lung function in both men and women. Smoking, physical activity and lung health, which are associated with CVD, are three important targets in the effort to mitigate disparities in cardiovascular health. This study, which has considerable individual and public health implications, provides evidence of the joint influence of smoking and sedentary lifestyle on lung function in AA adults. Since smoking and sedentary lifestyle both are negatively associated with healthy lungs, it is important that AAs who are at greater risk of CVD [47,48,49,50] and are known to have lower lung capacity than their white counterparts [23] refrain from smoking and adopt the practice of maintaining or increasing their physical activity. Although AAs might smoke less, and have a later age of onset for smoking than whites, the negative impact of both smoking and physical inactivity on their lung health, and, subsequently, on cardiovascular health may be substantial. There needs to be intensified efforts to prevent and/or intervene to address the hazards of smoking and physical inactivity that are both modifiable behaviors.

## References

[1] Sobol B., Herbert W., Emirgil C. (1974). The high incidence of pulmonary functional abnormalities in patients with coronary artery disease. Chest.

[2] Vestbo J., Lange P. (1991). Forced expiratory volume in 1 second (FEV1)—A respiratory physiological measurement of considerable prognostic value. Ugeskr. Laeger.

[3] Keys A., Aravanis C., Blackburn H., Djordjevic B.S., Dontas A.S, Fidanza F., Karvonen M.J., Menotti A., Taylor H.L. (1972). Lung function as a risk factor for coronary heart disease. Am. J. Public Health.

[4] Knuiman M.W., James A.L., Divitini M.L., Ryan G., Bartholomew H.C., Musk A.W. (1999). Lung function, respiratory symptoms, and mortality: Results from the Busselton Health Study. Ann. Epidemiol..

[5] Fulambarker A., Copur A.S., Javeri A., Jere S., Cohen M.E. (2004). References values for pulmonary function in Asian Indians living in the United States. Chest.

[6] Gordon-Larsen P., McMurray R.G., Popkin B.M. (1999). Adolescent physical activity and inactivity vary by ethnicity: The National Longitudinal Study of Adolescent Health. J. Pediatr..

[7] Kaczynski A.T., Manske S.R., Mannell R.C., Grewal K. (2008). Smoking and physical activity: A systematic review. Am. J. Health Behav..

[8] Kam S.W., Ping C., Hok C.L., Xin S.H., David S.C. (2000). The impact of heavy passive smoking on arterial endothelial function in modernized Chinese. J. Am. Coll. Cardiol..

[9] Ahmad A., Reza G., Alireza N., Amir G. (2011). Effects of aerobic exercise on lung function in overweight and obese students. Tanaffos.

[10] Garcia-Aymerich J., Lange P., Benet M., Schnohr P., Anto J. (2007). Regular physical activity modifies smoking-related lung function decline and reduces risk of chronic obstructive pulmonary disease. Am. J. Respir. Crit. Care Med..

[11] Katz P., Chen H., Omachi T.A., Gregorich S.E., Julian L., Cisternas M., Balmes J., Blanc P.D. (2011). The role of physical inactivity in increasing disability among older adults with obstructive airway disease. J. Cardiopulm. Rehabil. Prev..

[12] Jakes R.W., Day N.E., Patel B., Khaw K.T., Oakes S., Luben R., Welch A., Bingham S., Wareham N.J. (2002). Physical inactivity is associated with lower forced expiratory volume in 1 second: European Prospective Investigation into Cancer-Norfolk Prospective Population Study. Am. J. Epidemiol..

[13] Centers for Disease Control and Prevention (2008). Smoking-attributable mortality, years of potential life lost, and productivity losses—United States, 2000–2004. Morb. Mortal. Wkly. Rep..

[14] Centers for Disease Control and Prevention (2012). Health, United States, 2012: With Special Feature on Emergency Care.

[15] United States. Public Health Services (1998). Tobacco Use Among U.S. Racial/Ethnic Minority Groups—African Americans, American Indians and Alaska Natives, Asian Americans and Pacific Islanders, and Hispanics. The Report of the Surgeon General.

[16] Okuyemi K.S., Richter K.P., Ahluwalia J.S., Mosier M.C., Nazir N., Resnicow K. (2002). Smoking reduction practices among African American smokers. Nicotine Tob. Res..

[17] Markewitz B.A., Owens M.W., Payne D.K. (1999). The pathogenesis of chronic obstructive pulmonary disease. Am. J. Med. Sci..

[18] Thorn J., Bjorkelund S., Bengtsson C., Guo X., Lissner L., Sundh V. (2007). Low socio-economic status, smoking, mental stress and obesity predict obstructive symptoms in women, but only smoking also predicts subsequent experience of poor health. Int. J. Med. Sci..

[19] Lind E., Joens-Matre R.R., Ekkekakis P. (2005). What intensity of physical activity do previously sedentary middle-aged women select? Evidence of a coherent pattern from physiological, perceptual, and affective markers. Prev. Med..

[20] Siddiqi Z., Tiro J.A., Shuval K. (2011). Understanding impediments and enablers to physical activity among African American adults: A systematic review of qualitative studies. Health Educ. Res..

[21] Kriska A. (2000). Ethnic and cultural issues in assessing physical activity. Res. Q. Exercise Sport.

[22] Kim J.S., Bramlet M.H., Wright L.K., Poon L.W. (1998). Racial differences in health status and health behaviors of older adults. Nurs. Res..

[23] Adams-Campbell L.L., Rosenberg L., Washburn R.A., Rao R.S., Kim K.S., Palmer. J. (2000). Descriptive epidemiology of physical activity in African-American women. Prev. Med..

[24] Chang J.E., Lee J.H., Kim M.K., Kim S.J., Kim K.H., Park J.S., Kim T.H., Kim Y.I., Lee E.W., Kim J.O. (2006). Determinants of respiratory symptom development in patients with chronic airflow obstruction. Resp. Med..

[25] Agnarsson U., Thorgeirsson G., Sigvaldason H., Sigfusson N. (1999). Effects of leisure-time physical activity and ventilatory function on risk for stroke in men: The Reykjavik Study. Ann. Intern. Med..

[26] Wannamethee S.G., Shaper A.G., Ebrahim S. (1995). Respiratory function and risk of stroke. Stroke.

[27] Burchfiel C.M., Enright P.L., Sharp D.S., Chyou P.H., Rodriguez B.L., Curb J.D. (1997). Factors associated with variations in lung function among elderly Japanese-American men. Chest.

[28] Twisk J.W., Staal B.J., Brinkman M.N., Kemper H.C., van Mechelen W. (1998). Tracking of lung function parameters and the longitudinal relationship with lifestyle. Eur. Respir. J..

[29] Camoes M., Lopes C. (2008). Factors associated with physical activity in the Portuguese population. Rev. Saude Publica..

[30] Fuqua S.R., Wyatt S.B., Sarpong D., Henderson F.R., Cunningham M.F., Taylor H.A. (2005). Recruiting African-American research participation in the Jackson Heart Study: Methods, response rates, and sample description. Ethn. Dis..

[31] Taylor H.A., Wilson J.G., Jones D.J., Sarpong D.F., Srinivasan A., Garrison R.J, Nelson C., Wyatt S.B. (2005). Toward resolution of cardiovascular health disparities in African Americans: Design and methods of the Jackson Heart Study. Ethn. Dis..

[32] Dubbert P.M., Carithers T., Ainsworth B.A., Taylor H.A., Wilson G., Wyatt S.B. (2005). Physical activity assessment methods in the Jackson Heart Study. Ethn. Dis..

[33] Payne T.J., Wyatt S.B., Mosley T.H., Dubbert P.M., Guiterrez-Mohammed M.L., Calvin R.L., Taylor H.A., Williams D.R. (2005). Sociocultural methods in the Jackson Heart Study: Conceptual and descriptive overview. Ethn. Dis..

[34] Carithers T., Dubbert P.M., Crook E., Davy B., Wyatt S.B., Bogle M.L., Taylor H.A., Tucker K.L. (2005). Dietary assessment in African Americans: Methods used in the Jackson Heart Study. Ethn. Dis..

[35] Carithers T.C., Talegawker S.A., Rowser M.L., Henry O.R., Dubbert P.M., Bogle M., Taylor H.A., Tucker K.L. (2009). Validity and calibration of food frequency questionnaires used with African American adults in the Jackson Heart Study. J. Am. Diet. Assoc..

[36] Carpenter M., Crow R., Steffes M., Rock W., Heilbraun J., Evans G., Skelton T., Jensen R., Sarpong D. (2004). Laboratory, reading center, and coordinating center data management methods in the Jackson Heart Study. Am. J. Med. Sci..

[37] Wilson J.G., Rotimi C.N., Ekunwe L., Royal C.D., Crump M.E., Wyatt S.B., Steffes M.W., Adeyemo A., Zhou J., Taylor H.A. (2005). Study design for genetic analysis in the Jackson Heart Study. Ethn. Dis..

[38] Hankinson J., Odencrantz J., Fedan K. (1999). Spirometric reference values from a sample of the general U.S. population. Am. J. Resp. Crit. Care Med..

[39] Smitherman T., Dubbert P., Grothe K., Sung J.H., Kendzor D.E., Reis J.P., Ainsworth B.E., Newton R.J., Lesniak K.T., Taylor H.A. (2009). Validation of the Jackson Heart Study Physical Activity Survey in African Americans. J. Phys. Act. Health..

[40] Jacobs D.R., Nelson E., Dontas A.S., Keller J., Slattery M.L., Higgins M. (1992). Are race and sex differences in lung function explained by frame size? The CARDIA Study. Am. J. Respir. Dis..

[41] Harik-Khan R.I., Fleg J.L., Muller D.C., Wise R.A. (2001). The effect of anthropometric and socioeconomic factors on the racial difference in lung function. Am. J. Respir. Crit. Care Med..

[42] Higgins M., Keller J.B., Wagenknecht L.E., Townsend M.C., Sparrow D., Jacobs D.R., Hughes G. (1991). Pulmonary function and cardiovascular risk factor relationships in black and in white young men and women. The CARDIA Study. Chest.

[43] Tockman M.S., Comstock G.W. (1989). Respiratory risk factors and mortality: Longitudinal studies in Washington County, Maryland. Am. Rev. Respir. Dis..

[44] Tabak C., Spijkerman A.M., Verschuren W.M., Smit H.A. (2009). Does education level influence lung function decline (Doetinchem Cohort Study)?. Eur. Respir. J..

[45] Huxley R.R., Yatsuya H., Lutsey P.L., Woodward M., Alonso A., Folsom A.R. Impact of age at smoking initiation, dosage, and time since quitting on cardiovascular disease in African Americans and whites. Am. J. Epidemiol..

[46] Verbanck S., Schuermans D., Meysman M., Paiva M., Vincken. W. (2004). Noninvasive assessment of airway alterations in Ssokers. Am. J. Respir. Crit. Care Med..

[47] Ferrucci L., Izmirlian G., Leveille S., Phillips C.L., Corti M.C., Brock D.B., Guralnik J.M. (1999). Smoking, physical activity, and active life expectancy. Am. J. Epidemiol..

[48] Watkins L.O. (2004). Epidemiology and burden of cardiovascular disease. Clin. Cardiol..

[49] Mensah G.A., Mokdad A.H., Ford E.S., Greenlund K.J., Croft J.B. (2005). State of disparities in cardiovascular health in the United States. Circulation.

[50] Hozawa A., Folsom A., Sharrett A.R., Chambless L.E. (2007). Absolute and attributable risks of cardiovascular disease incidence in relation to optimal and borderline risk factors: Comparison of African American with white subjects—Atherosclerosis Risk in Communities Study. Arch. Intern. Med..

